# Metabolic Patterns of Flavonoid and Its Key Gene Expression Characteristics of Five Cultivars of *Tulipa gesneriana* during Flower Development

**DOI:** 10.3390/plants13030459

**Published:** 2024-02-05

**Authors:** Shu Li, Jing Chen, Xueying Guo, Xin Li, Qiang Shen, Xueqing Fu, Dongqin Tang

**Affiliations:** 1 School of Design, Shanghai Jiao Tong University, Shanghai 200240, China; 2 Instrumental Analysis Center, Shanghai Jiao Tong University, Shanghai 200240, China; 3 Shanghai Flower Port Enterprise Development Co., Ltd., Shanghai 200003, China

**Keywords:** *Tulipa gesneriana*, anthoxanthins, anthocyanin, flavonoid, key structural gene

## Abstract

Flower color is one of the most important ornamental traits of tulips (*Tulipa gesneriana*). Five typical tulip cultivars were selected to identify the flavonoid components and analyze their key gene expression in their tepals. Firstly, after preliminary determination of the pigment type, the flavonoids were identified by UPLC-Q-TOF-MS. A total of 17 anthoxanthins were detected in the five cultivars. The total anthoxanthin content in the white tulip and the red tulip showed a similar decreasing trend, while an increasing trend was observed in the black tulip. Similarly, a total of 13 anthocyanins were detected in five tulip cultivars. The black tulip contained the largest number of anthocyanins, mainly delphinidin derivatives (Dp) and cyanidin derivatives (Cy). The total anthocyanin content (TAC) in the orange, red, and black cultivars was higher than that in the white and yellow cultivars and presented an overall increase trend along with the flower development. *TgCHS*, *TgFLS*, *TgF3H*, *TgF3′H*, *TgF3′5′H,* and *TgDFR*, as key structural genes, were involved in the flavonoid synthesis pathway, and the expression patterns of these genes are basically consistent with the components and accumulation patterns of flavonoids mentioned above. Taken together, the flower color in tulips was closely related to the composition and content of anthocyanins and anthoxanthins, which were indeed regulated by certain key structural genes in the flavonoid pathway.

## 1. Introduction

Flower color is one of the phenotypes of plant adaptation evolution and is also a key indicator for evaluating the ornamental value of garden plants. With the increasing number of horticultural cultivars, plants become more and more colorful. The modulation of flower color is influenced by an array of factors, of which the primary contributors are both the type and content of pigments. Natural pigments are divided into three major categories: flavonoids, carotenoids, and betalains. Among them, betalains are very rare in plants, while flavonoids and carotenoids are widely distributed in plants. The ultimate observable hue is attributed to the blend of flavonoids housed in the vacuoles and carotenoids located within the plastids (chromoplasts), as exemplified by numerous *Viola tricolor* and *Capsicum annuum* [1,2]. An increasing body of research underscores that flavonoid compounds constitute the most vital pigments in the majority of plants. As we know, flavonoids have a decisive effect on the color of a variety of garden plants, resulting in various flower colors, such as yellow, orange, red, blue, purple, and other colors. For instance, in Chinese narcissus, the hue, brightness, and vividness of their petals present a significant correlation with flavonoids [3]. In the iris, delphinidin contributes to the flower color formation of purple iris (*Iris dichotoma*), while the flower color is mainly determined by pelargonidin in orange iris (*I. domestica*) [4]. In freesias, the petal color was closely related to the composition and content of anthoxanthins and anthocyanins [5]. In *Cymbidium ensifolium*, purple-red and red tepals contain a lot of anthocyanins, including cyanidin, pelargonin, and paeoniflorin [6]. While the flower color of *Carthamus tinctorius* changed from yellow to red during flower development, the changes in flavonoid biosynthesis were significant [7].

Up to date, the flavonoid biosynthesis has been well clarified, and the structural genes, including *CHS*, *FLS*, *F3′H*, *F3′5′H*, *DFR*, and *3GT*, i.e., encode key enzymes to control the synthesis of corresponding flavonoids, thereby causing color differentiation in plants [8,9]. Chalcone synthase (CHS) is the first key enzyme in flavonoid biosynthesis; it catalyzes the formation of chalcone, which is a precursor of many flavonoids in the later stage. Subsequently, more flavonoids are synthesized through several important branching pathways, which are regulated by specific key structural genes [10], in which two cytochrome P450s, named *F3′H* and *F3′5′H*, play key roles in the pathway. *F3′H* can promote the synthesis of cyanidin and change the flower color from red to magenta. While *F3′5′H* can promote the synthesis of delphinidin and change the flower color from purple to blue, it is also called the blue gene. In the absence of *F3′H* and *F3′5′H*, the plant will promote the synthesis of pelargonidin to change the flower color from orange to red. For instance, Shimada reported that the products of TG1 and AK14 in petunia produced differences in *F3′5′H* activity, thereby changing anthocyanin synthesis. Brugliera et al. found that *F3′H* transcription in *Chrysanthemum* × *morifolium* is most abundant in the early stages of flower development, and as the flower blooms, its transcription levels decrease [11]. *DFR*, an essential gene within this metabolic pathway, also assumes a significant function in regulating flower color. The expression level of *DFR* is responsible for the light yellow or orange-red color of carnation. Inhibiting the *DFR* expression in *Matthiola incana* changed the flower color from blue to a light blue or white. In the petals of *Tulipa fosteriana*, the red pigment accumulation was positively related to the expression levels of *TfDFR1* and *TfANS1* [12]. In freesia, *FhFLS1* and *FhFLS2* were also confirmed to be related to flavonoid biosynthesis so far [13].

Tulip (*Tulipa gesneriana*), a bulbous flower of the genus *Tulipa* in the family Liliaceae, is famous as a cut flower, potted flower, and landscape application around the world [14]. Most of the tulips used in modern gardens are horticultural cultivars, with bright colors, mostly pure colors such as yellow, white, red, and purple and some cultivars are complex colors. Tulip pigments mainly include two major categories: carotenoids and flavonoid compounds; the former usually makes tulips yellow, and the latter is considered the most common pigment in tulips with various flower colors, which can be further divided into anthocyanins and anthoxanthins. According to previous studies, 13 anthocyanins and 3 flavonols were identified in some tulips [15]. For instance, Torskangerpoll reported that four kinds of anthocyanins were detected in orange tulips [16]. Later, 5 anthoxanthins were identified in the flowers of 17 species and 25 cultivars of tulip. Yamagishi identified two new anthocyanins in the dark purple tulips and conducted a correlation analysis between flower color with anthocyanins, and its acetyl derivatives were the main cause of its purple color [17]. With the continuous breeding of tulips, new tulip cultivars with more colored flowers were emerging one after another, which may be accompanied by the production of new metabolic substances. Meanwhile, the study of the mechanism of flower color formation is relatively rare in tulips. Hence, it is necessary to more accurately identify its pigment components and explore the relationship between its metabolic patterns and flower color presentation. In this study, we identified the flavonoid metabolites, studied their accumulation patterns in tulip tepals, and analyzed the expression characteristics of key structural genes in the flavonoid synthesis pathway. The purpose is to explore the relationship between flavonoid metabolism and its gene expression with tulip flower color. These findings can lay a scientific foundation for the subsequent analysis of the mechanism of flower coloration in tulips and provide more evidence for the study of flower color in ornamental plants.

## 2. Results

### 2.1. Preliminary Identification of Anthocyanidin Types in Tulips

The color reaction results of five tulip cultivars are shown in Table 1. In the petroleum ether test, tulip ‘Starlight’ (SL), ‘Orange Emperor’ (OE), and ‘Parade’ (PD) showed different degrees of yellow, suggesting various carotenoids in their tepals. White ‘Purissima’ (PM) and ‘Queen of Night’ (QN) were both colorless, their tepals may contain little or no carotenoid. PD and QN, respectively, showed orange and red in the hydrochloric acid test, showing that their tepals contained anthocyanins, while the other cultivars showed colorless or light yellow, suggesting little or no anthocyanin in their tepals. In the ammonia test, QN showed yellow-brown; it might contain more flavanol compounds. The other cultivars had different degrees of yellow; they all contained flavones and flavonols, though the contents might vary.

### 2.2. Carotenoid Content in Tulips

**Figure 1:**
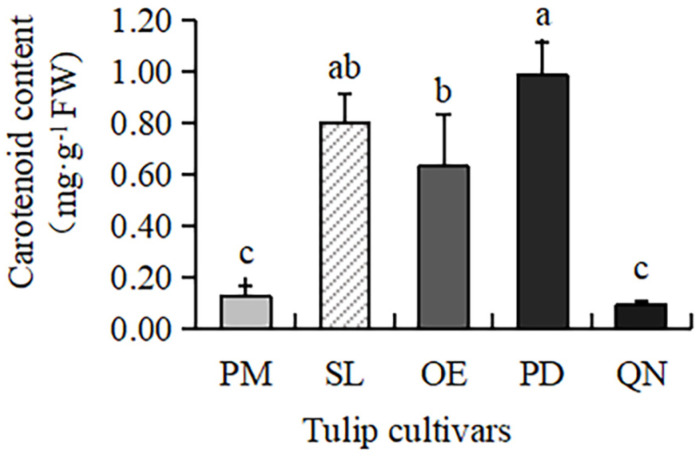
Carotenoid contents in 5 tulip cultivars. Different letters indicate significant differences by Duncan’s test (*p* < 0.05).

Carotenoids were present in all five tulip cultivars (Figure 1), but the content varied greatly, ranging from 0.09 to 0.98 mg·g^−1^ FW. The highest content was observed in PD, followed by SL and OE. A much lower content was present in QN and PM, only 0.09 mg·g^−1^ FW and 0.12 mg·g^−1^ FW, respectively. This result was consistent with the petroleum ether test, proving that carotenoids mainly existed in red, yellow, and orange tulip cultivars, while white and black cultivars had very few carotenoids.

### 2.3. Qualitative Analysis of Flavonoid in Tepals of Five Tulip Cultivars

#### 2.3.1. Identification of the Components of Anthoxanthins

**Figure 2:**
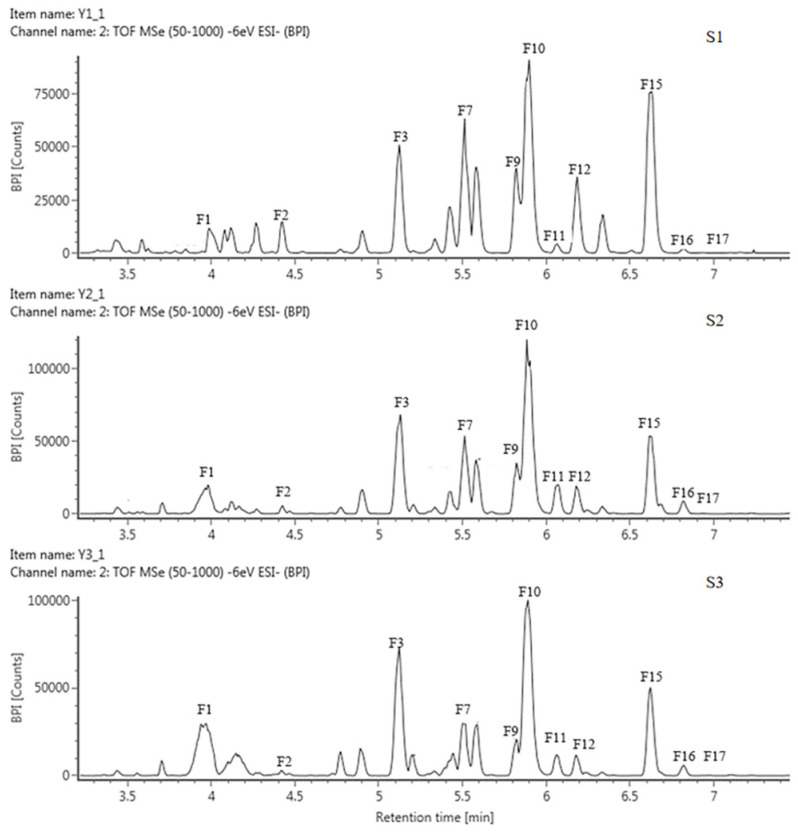
HPLC chromatogram of anthoxanthins in tepals at 3 flower development stages in QN.

Based on the retention time (RT) and mass-to-charge ratio (*m*/*z*) of the molecular ion (MI) and the fragment ion (FI), anthoxanthin components, including 12 flavonols and 5 flavones, were identified and then labeled as F1–F17, respectively (Table 2, Figure 2 and Figure S1). The 3 positions of the C-ring and the 7 positions of the A-ring of the glycoside element of flavonoids were usually the most susceptible positions for glycosyl substitution. The relative abundance of glycosidic ions [A − H]^+^ and [A − 2H]^+^ was the basis for determining the position of the glycosyl linkage. If the glycosylation was at position 3, the relative abundance of [A − 2H]^+^ was greater than that of [A − H]^+^. Instead, glycosylation occurred at position 7.

The FIs of components F1, F3, F5, F7, F8, F9, F10, and F12 all had an *m*/*z* of 301; hence, they were identified as quercetin derivatives. For F1, MI *m*/*z* was 610 [M − H]^−^, and the molecular weight difference between MI and FI was 309, corresponding to one molecule of neohesperidoside, so F1 was presumed to be quercetin 3-O neohesperidoside. The FIs of F3 were *m*/*z* 591 [Y0^−^] and m/z 301 [Y0^−^]; the loss of 164 and 294 in F3 corresponded to galactose and sambubiose, so it was identified as quercetin-3-galactose-sambubiose. The MI of F5 was *m*/*z* 625 [M − H]^−^, lost 324 by comparing with FI, which may be sophoroside, laminarabiose, or gentiobiose, so F5 was presumed as quercetin-3-diglucoside, The MI of F7, F8, F9 and F10 were all *m*/*z* 610 [M − H]^−^, the missing 308 corresponded to one molecule of rutinoside, suggesting that they were quercetin-rutinoside isomers, where F10 was directly identified as rutin (quercetin-3-rutinoside) by the standard. The MI of F12 was *m*/*z* 463 [M − H]^−^, 162 corresponding to one glucoside, so it was identified as quercetin-3-glucoside.

The FI of the components F2, F4, and F17 was *m*/*z* 285 [Y0^−^], showing that they were all kaempferol derivatives. F2 had a difference of 176 between MI and FI, which corresponded to one glucuronide. Therefore, it was deduced to be kaempferol 3-glucuronide. The MI of F4 and F17 were both *m*/*z* 447 [M − H]^−^, with a difference of 162 from FI, suggesting that they were the same as the kaempferol glycoside tautomers linked to a molecule of hexose. Based on the previous publication, F4 was identified as kaempferol 3-O-galactoside and F17 was kaempferol 3-O-glucoside.

The FI of F16 was *m*/*z* 315 [Y0^−^], corresponding to the isorhamnetin derivative; the difference of 308 between MI and FI corresponded to rutinoside. Therefore, it was assumed to be isorhamnetin 3-rutinoside.

Except for the above 12 flavonols, 5 flavones were identified, including F6, F11, F13, F14, and F15. Among them, the FI of F6 was *m*/*z* of 271, showing that it was a naringenin derivative, with a MI of *m*/*z* of 433 [M − H]^−^, the missing 162 was corresponding to one glucose, so it was presumed to be naringenin 3-glucoside. The FIs of F11 and F14 were both *m*/*z* 269 [Y0^−^], corresponding to apigenin derivatives, the difference was 162 and 308 between MI and FI, respectively, usually corresponding to one glucoside and one rutinosides, so they were identified as apigenin 7-glucoside and apigenin 7-rutinoside, respectively. The FI of F13 was *m*/*z* 302 [Y0^−^], suggesting it belongs to hesperidin derivative, a difference of 162 between its MI (*m*/*z* 463 [M − H]^−^) and FI, corresponding to one glucoside, so F13 was then presumed as hesperedin 7-O-glucoside. The FI of F15 was *m*/*z* 270 [Y0^−^], belonging to the baicalin derivative, the difference with the MI corresponds to two glucosides, so F15 was identified baicalin-7-diglucoside.

#### 2.3.2. Identification of the Components of Anthocyanins

**Figure 3:**
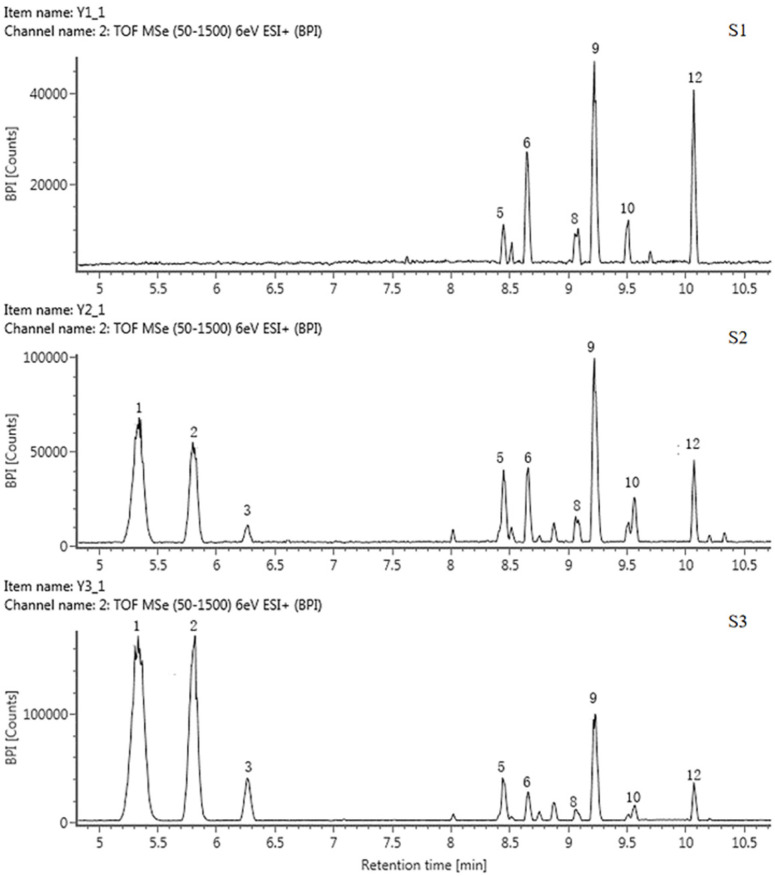
HPLC chromatogram of anthocyanins in tepals at 3 flower development stages in QN.

A total of 13 anthocyanins were identified and labeled as A1-A13 (Table 3, Figure 3 and Figure S2). Only A10 was identified as cyanidin 3-O-glucoside by the standard, and the rest of the components were presumed based on the mass spectrum data and references.

The FIs of components A1, A6, and A8 were all 303 ([Y0^+^]), suggesting that they were all delphinidin derivatives. The FIs of A9 and A12 were both 287 ([Y0^+^]), so they should be cyanidin aglycone. The FIs of A3 and A11 were both 271 ([Y0^+^]), which were pelargonidin aglycone, and the FI of A13 was 317, indicating that it was a petunidin derivative. The MI and FI of the A1 and A12 differed by 308, corresponding to one rutinoside, suggesting that they were both rutinoside derivatives. MI and FI of A6 and A9 differed by 132, corresponding to one arabinose, suggesting that these two components were arabinosylated. Subsequently, A1, A6, A9, and A12 were identified as delphinidin 3-rutinoside, delphinidin 3-arabinoside, cyanidin 7-arabinoside and cyanidin 3-rutinoside, respectively. The theoretical fragmentation values indicate that A8 was a bis-glycosidized substance; the FI values were *m*/*z* 464 [Y0]^+^ and *m*/*z* 303 [Y0]^+^; the loss of 147 and 162 corresponded to one rhamnose and one glucose, so A8 was presumed to be a delphinidin 3-rhamnoside 5-glucoside. Similarly, A2 was identified as cyanidin 3-rhamnoside 5-glucoside. A3, A4, and A11 were identified as pelargonidin 3-rutinoside, pelargonidin, and pelargonidin 3-rhamnoside, respectively. A13 was identified as petunidin 3-rutinoside.

Mass spectrometry (MS) data showed that, in these five tulip cultivars, none of them contained all the above 13 types of anthocyanins; they varied from 4 to 9 types of anthocyanins. Among them, QN contained the largest number of 9 anthocyanins, mainly delphinidin derivatives and cyanidin derivatives. OE and PD contained 5 and 6 anthocyanosides, which were mainly pelargonidin derivatives. Four and five anthocyanins were detected in PM and SL, which were mainly cyanidin derivatives.

### 2.4. Metabolic Patterns of Flavonoid in Tepals of Five Tulip Cultivars

#### 2.4.1. Metabolic Patterns of Anthoxanthins

**Figure 4:**
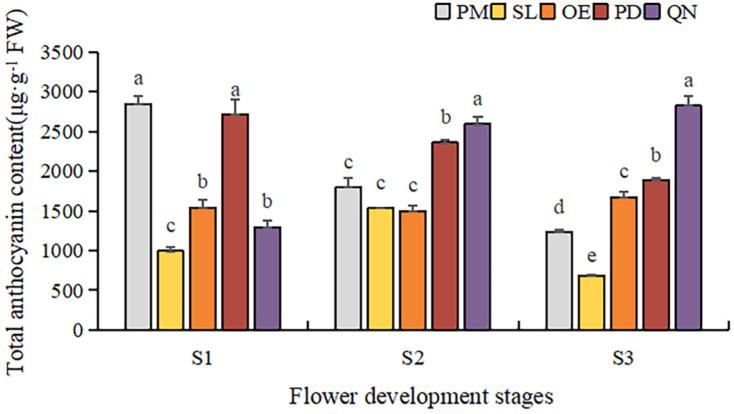
Total anthoxanthin contents in tulip tepals in different flower development stages. Different letters indicate significant differences in each stage by Duncan’s test (*p* < 0.05).

The accumulation patterns of anthoxanthins were different in each cultivar, along with flower development stages (Figure 4). The total anthoxanthin content (TAX) in PM and PD showed a similar decreasing trend; TAX at S3 was only 0.43- and 0.69-fold of that at S1. While an increasing trend was observed in QN, its TAX peaked at S3 (2822.7 μg·g^−1^ FW), 2.2 times that at S1. TAX in OE and SL was relatively lower than above 3 cultivars. In SL, TAX first increased and then decreased, peaking at S2 (1532.8 μg·g^−1^ FW), which was 2.3 times that at S3. TAX in OE was between 1500 and 1700 μg·g^−1^ FW; only a slight increase at S3 was present. At each stage, the TAX of the five tulips also varied greatly. At S1, the highest TAX was observed in PM, 2.2 times the lowest in QN. At S3, QN had the highest anthoxanthin, 4.1 times the lowest TAX (680.7 μg·g^−1^ FW) in SL.

#### 2.4.2. Metabolic Patterns of Anthocyanins

**Figure 5:**
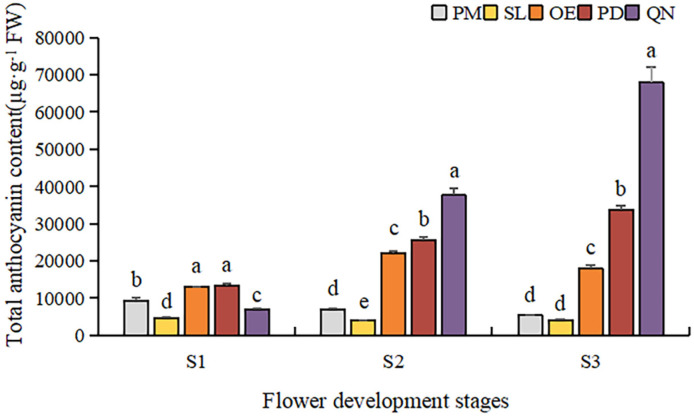
Total anthocyanin contents in tulip tepals at different flower development stages. Different letters indicate significant differences in each stage by Duncan’s test (*p* < 0.05).

By comparing the total anthocyanin content (TAC) of five tulip cultivars at different stages, a significant difference was observed (Figure 5). Overall, TAC in orange, red, and black tulips was significantly higher than that in yellow and white tulips and showed an upward trend along with the flower development. At S1, OE and PD had a significantly higher TAC than the other three cultivars. As the flower bloomed, the TAC of PD and QN gradually increased, and QN was significantly higher than the other four cultivars at S3. The highest TAC (67,939.6 μg·g^−1^ FW) was detected in QN at S3, followed by that in PD and OE, corresponding to 31,784.1 μg·g^−1^ FW and 18,042.1 μg·g^−1^ FW, respectively. Although certain anthocyanins were detected in the white tulip (PM) and yellow tulip (SL), their TAC was very low. At S3, only 334.3 μg·g^−1^ FW in PM was observed, only corresponding to 0.4% of QN at this stage. And the TAC detected in SL was even lower than that in PM at each stage.

**Figure 6:**
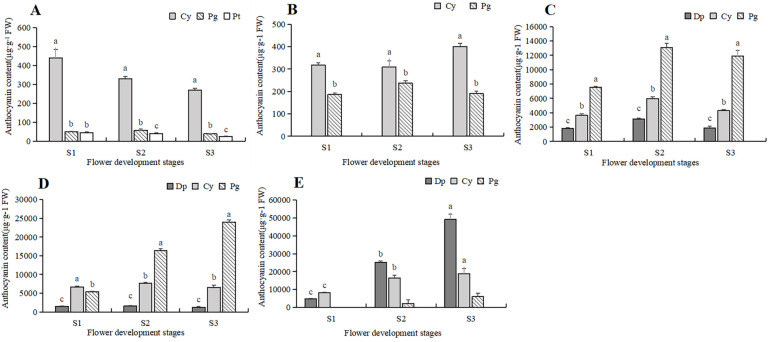
Anthocyanins contents in 5 tulip cultivars. Different letters indicate significant differences in each stage by Duncan’s test (*p* < 0.05). (**A**) PM, (**B**) SL, (**C**) OE, (**D**) PD, and (**E**) QN.

To further clarify the difference in anthocyanin content, a comparative analysis was conducted on the content of various anthocyanin components in five tulip cultivars, and the relationship between the anthocyanin and the flower color of the tulip was then analyzed (Figure 6). In the white tulip PM, the dominant component of anthocyanin was cyanidin derivatives (Cy), accompanied by a very small amount of pelargonidin derivatives (Pg) and petunidin derivatives (Pt). These anthocyanins decreased along with the flower development process. In the yellow tulip SL, the anthocyanins were Cy and Pg, which also showed a decreasing trend. Three anthocyanins, Dp, Cy, and Pg, were detected in the orange tulip OE and the red tulip PD, and Pg was the dominant component at each stage. Basically, a similar accumulation pattern of these anthocyanins was observed in both cultivars. Pg showed an increasing trend during the flower development stages, while Cy and Dp first increased and then decreased, which were significantly lower than Pg, especially at S2 and S3. In the black tulip QN, delphinidin derivatives (Dp), cyanidin derivatives (Cy), and pelargonidin derivatives (Pg) were all detected, and they all showed an increasing trend along with flower development. Meanwhile, Dp was significantly higher than Cy and Pg, and its proportion gradually increased, accounting for 72.2% of TAC at S3.

#### 2.4.3. Metabolic Patterns of Total Flavonoids

**Figure 7:**
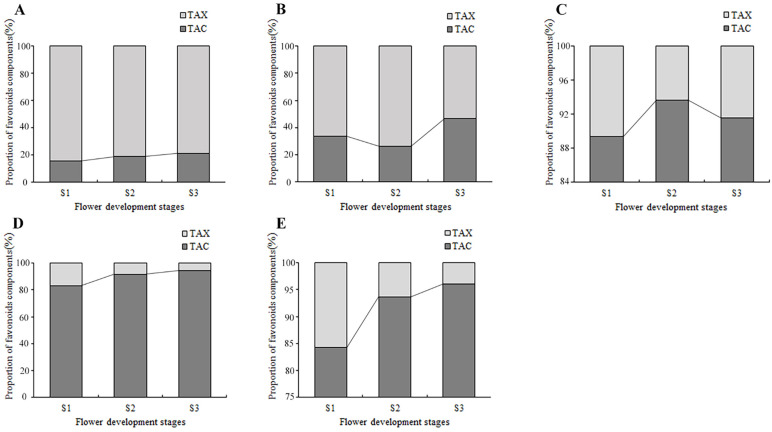
The accumulation of flavonoids in tepals at flower development stages. (**A**) PM, (**B**) SL, (**C**) OE, (**D**) PD, and (**E**) QN.

By counting the contents of anthoxanthin and anthocyanin at each flower development stage, the dynamic change of their proportion in the total flavonoids was obtained (Figure 7). In PM and SL, the anthoxanthins were always much higher than that of anthocyanins at all stages, and the highest percentage of TAX was 68.3% and 47.5% higher than the highest percentage of TAC, respectively. In tulips OE, PD, and QN, the anthocyanins accounted for more than 80% of the total flavonoid content, much higher than the anthoxanthins. The proportion of anthocyanin in the total flavonoid content of PD and QN increased continuously, reaching 94.7% and 96% at S3. While the proportion of anthocyanin in OE first increased and then decreased, peaked at S2.

### 2.5. Expression of Key Structural Genes in the Flavonoid Synthesis Pathway

**Figure 8:**
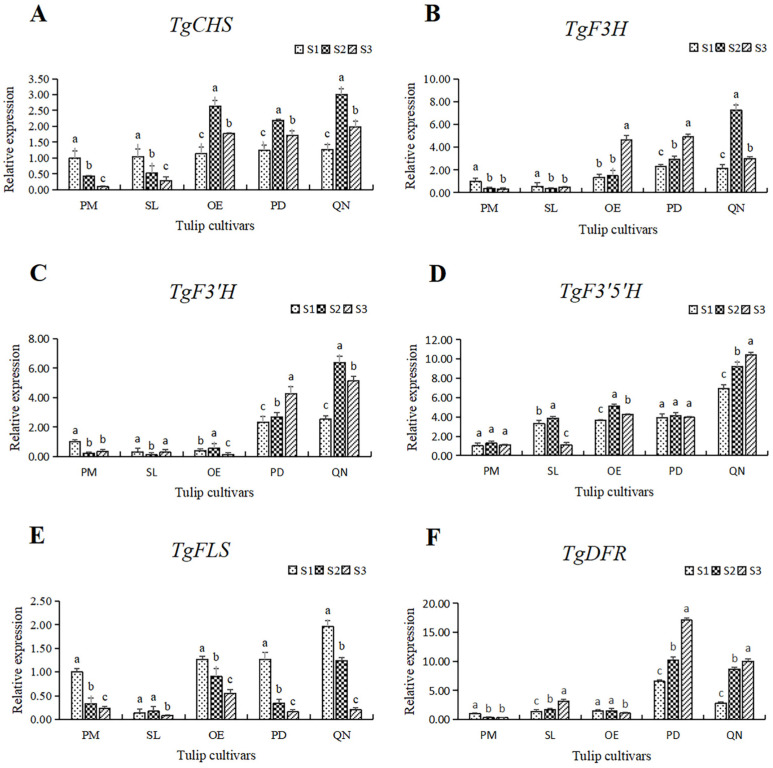
The relative expression of six structural genes involving the flavonoid synthesis pathway in 5 tulip cultivars. Different letters indicate significant differences at each stage by Duncan’s test (*p* < 0.05). (**A**) *TgCHS*, (**B**) *TgF3H*, (**C**) *TgF3′H*, (**D**) *TgF3′5′H*, (**E**) *TgFLS,* and (**F**) *TgDFR*.

In order to further understand the molecular mechanism of flavonoid synthesis in tulip tepals, six key structural genes in this pathway were selected for expression pattern analysis. The expression of these genes varied within 5 tulip cultivars, and their expression also differed at different stages in individual cultivars (Figure 8).

The expression of *TgCHS* in both PM and SL showed a continuous decreasing trend. While in OE, PD, and QN, the expression of *TgCHS* increased first, followed by a decrease, reaching its highest level at S2. At all stages, the expression level of *TgCHS* in the black tulip QN was higher than that of the other four cultivars, and it peaked at S3, corresponding to 19.8 times that of the white tulip PM at S3. In general, the expression of *TgCHS* in the orange, red, and black tulips was higher than that in the white and yellow tulips, except at S1 (Figure 8A).

Among the five tulips, the *TgF3H* expression in the white and yellow tulips was significantly lower than that in the other three cultivars at all stages (Figure 8B). The expression level of *TfF3H* in PM and SL was quite low and showed a continuous decrease along with flower development. In OE and PD, the expression of *TgF3H* showed an upward trend, peaking at S3, corresponding to 3.54 and 2.12 times that at S1. The expression of *TgF3H* in QN first increased and then decreased. The highest level of *TgF3H* was observed in QN at S2, corresponding to 24.2 times the lowest in PM at S3.

The expression level of *TgF3′H* in the white, yellow, and orange tulips was much lower than that in the red and black tulips (Figure 8C). In PM and SL, the expression of *TgF3′H* first decreased, followed by a slight increase. In OE, the expression of *TgF3′H* peaked at S2 and declined significantly at S3. An overall increase in the expression of *TgF3′H* was observed in PD and QN, and a significantly higher level of expression of *TgF3′H* was observed at S2 and S3 in both cultivars than that at S1. Among all samples, the highest level of *TgF3′H* was present in QN at S2, corresponding to 53.3 times the lowest level in SL.

The expression level and the change pattern of *TgF3′5′H* varied among five tulips (Figure 8D). In the white tulip PM, the expression was very low, and there was no significant change at all three stages, while a significantly higher level was observed and showed a continuous upward trend in the black tulip QN. In the other 3 cultivars, the expression of *TgF3′5′H* was similar at a moderate level, except for a significant decrease in SL at S3. The highest value observed in QN at S3 was 10.4 times the lowest value observed in PM at S1.

Unlike other genes, the expression of *TgFLS* showed a significant downward trend with flower development, with the highest and lowest values appearing at S3 and S1, respectively (Figure 8E). In SL, the *TgFLS* expression was significantly lower at S1 and S2 than that of other cultivars. The lowest expression was observed in SL at S3, only equivalent to 4.1% of the highest value in QN at S1.

A similar expression pattern of *TgDFR* with *TgF3′H* was observed (Figure 8F). The gene expression level in the white, yellow, and orange tulips was much lower than that in the red and black tulips, among all stages. A slight decrease was present in PM and OE, while a continuous increase was observed in SL, PD, and QN. The expression of *TgDFR* in PD was the highest at each stage, followed by QN.

## 3. Discussion

### 3.1. Flavonoid Is the Key Metabolite Regulating Tulip Flower Colors

In this study, carotenoids and flavonoids were detected in the tepals of 5 tulip cultivars with different flower colors, but the content of carotenoids was relatively low. Therefore, flavonoid substances are the main metabolites for flower pigmentation in tulips. By UPLC, a total of 30 flavonoids, including 17 anthoxanthins and 13 anthocyanins, were identified in five tulips, and the content and proportion of flavonoids were related to the flower color of the tulips.

The anthoxanthins in the tepals of the white and yellow tulips were much higher than the anthocyanins. It is already known that the hue range of flavonols generally ranges from colorless to light yellow. Therefore, higher TAX may be the reason for the light color of these two cultivars. The color of flowers is closely related to the types and content of anthocyanins. Previous studies have shown that the orange, red, blue, and purple colors in tulips are mostly regulated by anthocyanins. As shown above, in the orange, red, and black cultivars, TAC was much higher than TAX in their tepals and increased as flowers bloomed, which was similar to the results in *Lagerstroemia indica* [25]. Especially in the dark tulip QN, anthocyanins account for a decisive proportion of flavonoid substances after flower pigmentation. Similar results were also reported in other black flowers, such as *Cosmos atrosanguineus*, in which TAC in black cultivars was observed to be three to four times higher than that in the red cultivar [26].

Except for the content, the types of anthocyanins also affect the flower color of plants. In the flavonoid synthesis pathway, there are three branches for the synthesis of anthocyanins. Many plants often lack a certain branch, resulting in different anthocyanin metabolites, which then affect flower color [27]. Our previous research found that the main anthocyanin substances in red tulips were pelargonidin (Pg) and cyanidin (Cy) [28]. In the present study, it was found that Pg, delphinidin (Dp), and Cy were all detected in the orange, red, and black tulips. Furthermore, the accumulation of Cy derivatives and Pg derivatives in the orange and red tulips was much higher than that of Dp derivatives, confirming that Pg and Cy substances indeed made the tulip appear orange and red [29]. Unlike the previous two cultivars, in the black tulip QN, Dp had the highest proportion, accompanied by a moderate content of Cy and a very low proportion of Pg. Meanwhile, with the blooming of flowers, the proportion of Dp derivatives increased significantly, suggesting that the large accumulation of Dp was the main reason for the tulip showing dark purple to black color, which was also consistent with our previous study and other black flowers.

No malvidin, petunidin, or peonidin were detected in these tulips, which may be due to a lack of the methyltransferase required for their synthesis [30]. The absence of these enzymes could mean that the pathways for synthesizing malvidin, petunidin, and peonidin were incomplete or even absent. This could be a result of a variety of factors, such as a genetic mutation inhibiting enzyme production, downregulation of the related genes, or an evolutionary adaptation suited to the specific needs and environment of the tulip.

### 3.2. Key Structural Genes in the Flavonoid Pathways Regulating the Formation of Specific Flavonoids, thereby Affecting Flower Color in Tulips

Genes can influence flower color by affecting metabolites in the flavonoid metabolic pathways [31]. Since flavonoid is the main pigment in tulip flowers, six key structural genes involved in the pathway were selected to analyze their expression level by qRT-PCR. The expression patterns of these genes are consistent with the components and accumulation patterns of flavonoids mentioned above. CHS is the first key upstream synthesis enzyme in the pathway; it catalyzes the formation of chalcone, which is an important precursor for the subsequent synthesis of different flavonoids [32]. In the white and yellow tulips, a higher expression of *TgCHS* and *TgFLS* was mostly present at S1, revealing its main pigments, anthoxanthins, were synthesized at an early stage, which is consistent with the accumulation pattern of anthocyanin in the two cultivars. *F3H* is a key upstream synthesis gene for following the synthesis of anthocyanins. In the orange, red, and black tulips, their expression level was much higher than that in the white and yellow cultivars, corresponding to the accumulation pattern of their anthocyanins. *F3′5′H* and *F3′H* were two important branching genes of anthocyanins, regulating the formation of Dp and Cy, respectively. Studies have shown that the expression of *F3′5′H* in the flavonoid synthesis pathway is closely related to the accumulation of delphinidin in black flowers [33]. Here, in the black tulip, the high expression of *TgF3′5′H* leads to the synthesis of anthocyanins shifting to the Dp branch [34]. Similar to hyacinth, the anthocyanins in black ‘Black Baccara’ were mainly Dp, and the expression of *HoF3′5′H* was much higher than that of *HoF3′H* [35]. In orange and red tulips, relatively high Cy and Pg may be related to the expression of *TgF3′H* and *TgDFR* and then jointly influence the coloring of flowers.

## 4. Materials and Methods

### 4.1. Plant Materials

Five cultivars of *Tulipa gesneriana* were used as materials, including ‘Prussima’, ‘Starline’, ‘Orange Emperor’, ‘Parade’, and ‘Queen of Night’ (shorted for PM, SL, OE, PD, and QN, respectively), which were provided by Shanghai Flower Port Enterprise Development Co., Ltd., Shanghai, China.

**Figure 9:**
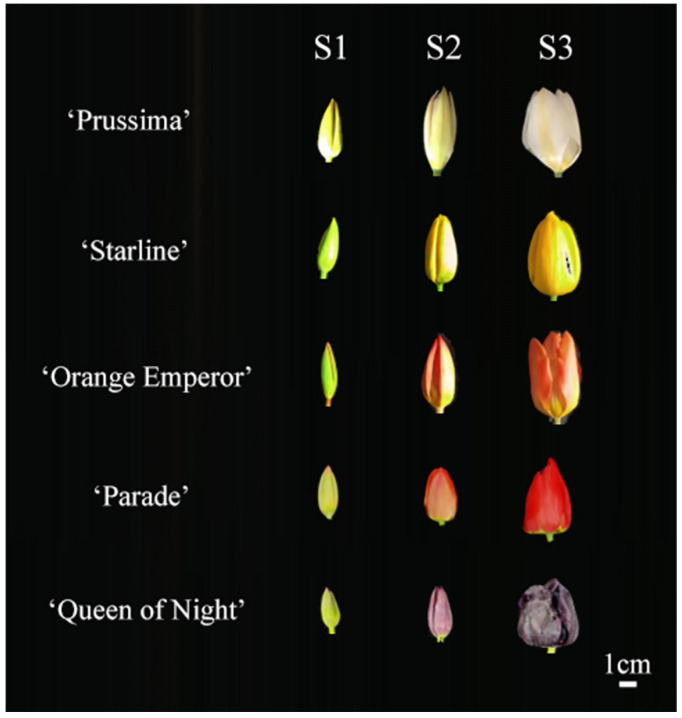
Flower phenotype of 5 tulip cultivars in three stages.

The bulbs were planted in the Modern Agricultural Engineering Training Center of Shanghai Jiao Tong University in October 2022. All samples were collected during the flowering period in 2023 spring. The flowers were divided into three stages according to the flower developmental process, including S1 (the green flower bud stage), S2 (the flower bud in the complete coloring stage), and S3 (the blooming stage) (Figure 9) [36]. The tepals of each stage were separated from the flowers and collected separately. Each sample was a mixture of tepals from five individual plants. Samples were immediately frozen in liquid nitrogen and then stored at −80 °C.

### 4.2. Methods

#### 4.2.1. Color Reactions of Tepals in Five Tulips

Color reaction was a commonly used method for preliminary determination of the type of pigment [37]. For each cultivar, 0.1 g of tulip tepals at S3 were placed into a mortar. Then, 5 mL of petroleum ether, 10% hydrochloric acid, and 30% ammonia were added to the mortar, and the tepals were quickly ground, filtered, and observed. All reaction solutions were photographed and recorded as color phenotypes under consistent indoor lighting conditions. Then, based on the color of the reaction solution, the type of pigment was preliminary determined in the five tulips, respectively [38].

#### 4.2.2. Extraction and Determination of Carotenoid in Five Tulips

Tepals at S3 of five tulips were taken for carotenoid quantitative analysis. Carotenoids were extracted by ethanol according to the method described in previous references [39,40]. Three portions of fresh tepals weighing 0.1 g were put into a mortar; a small amount of quartz sand and calcium carbonate powder, as well as 3 mL of 95% ethanol, were added and ground quickly; then the solution was placed in the dark for 10 min, filtered into 25 mL volumetric flasks, rinsed repeatedly with 95% ethanol until almost colorless, and finally fixed to 25 mL with 95% ethanol and shaken well. Using 95% ethanol as a blank-control group, 2 mL of extract was injected into a 1 cm aperture cuvette, and the absorbance of the carotenoid extracts of each sample of tulip tepals was measured by a UV-permittable spectrophotometer (BioTek, Winooski, VE, USA) at 470 nm, 649 nm, and 665 nm, respectively. The carotenoid content was calculated according to the following formula:

Carotenoid concentration (mg·L^−1^) was counted by the following formula:

$$c=\frac{1000A_{470}+811.7385A_{665}+2851.304A_{649}}{245}$$

Carotenoid content (mg·g^−1^·FW) was counted by the following formula:

M = (c × V)/(m × 1000)

where A_470_, A_649_, and A_665_ are the absorbance of extracts at 470 nm, 649 nm, and 665 nm, respectively; V is the volume of carotenoid extracts (25 mL); and m is the fresh tepal quantity (weight) for extracting carotenoids.

#### 4.2.3. Extraction and Determination of Flavonoids in Five Tulips

Here, 0.2 g of tepal powder was weighed in a centrifuge tube, then 2 mL of extraction solution (0.1% hydrochloric acid aqueous solution in 70% methanol) was added, followed by being vortexed for 30 s, the solution was extracted in ultrasonic oscillation for 40 min, then put in 4 °C refrigerator under dark for 24 h and shook every 6 h. After the above extraction, 1 mL of supernatant was taken to centrifuge for 20 min at 24 °C and 12,000 rpm, and 800 μL of supernatant was taken for subsequent analysis.

The qualitative and quantitative analysis of flavonoids in tulip tepals was performed by using ultra-high-performance liquid chromatography and VION ion mobility quadrupole time-of-flight mass spectrometry (UPLC-Q-TOF-MS) (Waters Cooperation, Milford, CT, USA). The analysis conditions were referred to in our previous research [5]. The conditions for the UPLC-Q-TOF-MS analysis were as follows: The chromatographic column used was a BEH C18 1.7 μm (2.1 × 100 mm). The mobile phase A was 0.1% formic acid, and B was 0.1% formic acid in acetonitrile. The elution gradient was established as: 0 min, 5% B; 3 min, 20% B; 10 min, 100% B; 12 min, 100% B; 15 min, 95% B; 20 min, 95% B. The flow rate was set to 0.4 mL/min, the sample injection volume was set at 1 μL, and the column temperature was maintained at 45 °C. As for the conditions for mass spectrometry, the data acquisition mode was MSE (low energy/high energy switching scan). The ion detection mode for the flavonols was electrospray negative ion scan mode (*m*/*z* 50–1000), and for the anthocyanins, it was electrospray positive ion scan mode (*m*/*z* 50–1000), with a scan speed of 0.2 s. The capillary voltage was 2 KV, and the cone voltage was 40 V. The atomization gas temperature was set to 450 °C, with an atomization gas flow rate of 900 L/h. The counter-blowing gas flow for the cone was 50 L/h, and the ion source temperature was 115 °C. The external standard method was used to quantify the flavonoids in tulip tepals [41]. The standards were dissolved in methanol and prepared into concentrations of 0.1, 0.5, 1, 5, 10, 50, 100, and 500 μg·mL^−1^ for constructing the standard curves. Rutin was chosen as a reference to identify anthoxanthins, and cyanidin-3-O-glucoside was chosen as a reference to identify anthocyanins. The results were calculated by fresh weight.

#### 4.2.4. Expression Analysis of Key Structural Genes in the Flavonoid Synthesis Pathway

The total RNA was isolated from each sample with OminiPlant RNA Kit (DNase I) following the instructions (CWBIO, Beijing, China). The first-strand cDNA for quantitative real-time PCR (qRT-PCR) was synthesized from total RNA using the PrimeScript RT Master Mix (TaKaRa, Shiga, Japan). The results of qRT-PCR were performed with ChamQ Μniversal SYBR qPCR Master Mix (Vazyme, Nanjing, China). The primers (Table S1) used in the qRT-PCR reaction were designed using the software Primer Premier 5 and synthesized by Shanghai Sangon Biotech, Shanghai, China. *TgActin* (GenBank NO. GQ340770) was used as the reference gene. Based on our previous transcriptome databases (BioProjects PRJNA815917 and PRJNA816321), six key enzyme genes in flavonoid biosynthesis pathway, including *TgCHS* (transcript_48901), *TgF3H* (transcript_51735), *TgF3′5′H* (transcript_41164), *TgF3′H* (transcript_40499), *TgFLS* (transcript_52943), and *TgDFR* (transcript_49202), were selected to analyze. The relative expression level of the selected genes was calculated according to the 2^−△△Ct^ method [42]. The expression of PM at S1 was set to 1, and the gene expression of other samples was normalized to this value.

#### 4.2.5. Statistical Analysis

Three biological replicates were included for all experiments. In the figures, the values were presented as the mean ± standard deviation (SD) of three replicates. IBM SPSS 14.0 software was used to analyze the significant difference by the Duncan test (*p* < 0.05).

## 5. Conclusions

A total of 17 anthoxanthins and 13 anthocyanins were detected in the tepals of five tulip cultivars in the present study. There were significant differences in the components and contents of anthoxanthins and anthocyanins among different cultivars, and the accumulation of these flavonoids varied among different cultivars at different flower developmental stages. The expression patterns of six key structural genes were consistent with the components and accumulation patterns of flavonoids mentioned above. Taken together, the differences in the composition and content of flavonoids are important reasons for the flower color formation in tulips. The key enzyme genes in the synthesis pathway indeed play an important role in the generation of different flavonoid substances, thereby affecting the presentation of flower color in tulips.

## Figures and Tables

**Table 1:** Results of color reaction in tepals of 5 tulip cultivars.

Cultivar	Petroleum Enter	10% HCI	NH_3_·H_2_O	Pigment Type
PM	Colorless	Colorless	Light-yellow	Flavonols
SL	Yellow	Colorless	Light-yellow	Carotenoids and flavonols
OE	Light-yellow	Light-yellow	Yellow	Carotenoids, anthocyanin, and flavonols
PD	Yellow	Orange	Yellow	Carotenoids, anthocyanin, and flavonols
QN	Colorless	Red	Dark-yellow	Anthocyanin and flavonols

**Table 2:** Anthoxanthins in tepals of five tulip cultivars.

No.	RT (min)	Molecular Ions (*m*/*z*)	Fragment Ions (*m*/*z*)	Identified Components	Cultivars	Reference for Identification
F1	3.96	610	301	Quercetin-3-O-neohesperidoside	SL, PD, QN	[18]
F2	4.41	461	285	Kaempferol-3-glucuronide	PM, SL, OE, PD, QN	[19,20]
F3	5.13	755	591,301	Quercetin-3-galactose-sambubiose	PM, QN	[19]
F4	5.24	447	285	Kaempferol-3-O-galactoside	OE	[21]
F5	5.29	625	301	Quercetin 3- diglucoside	PM, PD	[22]
F6	5.43	433	271	Naringenin 3-glucoside	PM, SL	[18,19]
F7	5.53	609	301	Quercetin derivative	SL, QN	[20,22]
F8	5.7	609	301	Quercetin derivative	PM, SL, PD	[22]
F9	5.82	609	301	Quercetin derivative	QN	[22]
F10	5.91	609	301	Rutin	PM, SL, OE, PD, QN	Std *
F11	6.01	431	269	Apigenin 7-glucoside	PM, SL, PD, QN	[19]
F12	6.18	463	301	Quercetin 3-glucoside	PM, SL, PD, QN	[22]
F13	6.38	463	302	Hesperetin 7-O-glucoside	SL, OE	[23]
F14	6.52	577	269	Apigenin-7-rutinoside	SL, OE, PD	[18]
F15	6.64	593	270	Baicalin-7-diglucoside	PM, SL, OE, PD, QN	[18]
F16	6.83	623	315	Isorhamnetin-3-rutinoside	PM, SL, OE, PD, QN	[20,21,23]
F17	6.94	447	285	Kaempferol-3-O-glucoside	PM, SL, OE, PD, QN	[22,23]

* Note: Std: The component was identified by the standard compound.

**Table 3:** Anthocyanins in tepals of five tulip cultivars.

No.	RT (min)	Molecular Ions (*m*/*z*)	Fragment Ions (*m*/*z*)	Tentative Identification	Cultivars	Reference for Identification
A1	5.34	611	303	Delphinidin 3-rutinoside	QN	[16,24]
A2	5.81	595	448, 287	Cyanidin 3-rhamnoside 5-glucoside	QN	[16]
A3	6.27	579	271	Pelargonidin 3-rutinoside	OE, PD, QN	[16]
A4	7.65	271	/	Pelargonidin	PD	[16]
A5	8.45	465	303	Delphinidin3-O-glucoside	QN	[16]
A6	8.66	435	303	Delphinidin 3-arabinoside	QN	[24]
A7	9.14	433	271	Pelargonidin 3-glucoside	PM, SL, OE, PD	[16,24]
A8	9.23	611	464, 303	Delphinidin 3-rhamnoside 5-glucoside	OE, PD QN	[24]
A9	9.49	419	287	Cyanidin 7-arabinoside	SL, QN	[16,24]
A10	9.56	449	287	Cyanidin 3-rutinoside	PM, SL, QN	Std *
A11	9.69	417	271	Pelargonidin 3-rhamnoside	SL, OE, PD	[16]
A12	10.07	595	287	Cyanidin 3-pelargonidin	PM, SL, OE, PD, QN	[24]
A13	10.25	625	317	Petunidin 3-rutinoside	PM	[16,24]

* Note: Std: The component was identified by the standard compound.

## Data Availability

The data that support the findings of this study are available in the insert article or the Supplementary Materials here.

## Supplementary Materials

The following supporting information can be downloaded at: https://www.mdpi.com/article/10.3390/plants13030459/s1, Table S1. The sequences of the primers; Figure S1. HPLC chromatogram of anthoxanthins in tepals at 3 flower development stages. (A) PM, (B) SL, (C) OE, (D) PD; Figure S2. HPLC chromatogram of anthocyanins in tepals at 3 flower development stages. (A) PM, (B) SL, (C) OE, (D) PD.
