# Supplementary material for: Metabolic Patterns of Flavonoid and Its Key Gene Expression Characteristics of Five Cultivars of Tulipa gesneriana during Flower Development
Source: Plants (Basel). 2024 Feb 5;13(3):459. doi: 10.3390/plants13030459 (PMC10857304; doi:10.3390/plants13030459)
Supplement: Supplementary file 1 [file plants-13-00459-s001.zip › plants-2805519-supplementary.pdf]

Supplementary Materials:

Table S1. The sequences of the primers

Primers	Sequence（5′-3′）
CHS-Q-F	GCTCAGAAATCACCGCAGTTAC
CHS-Q-R	CTCGATAGCCAGATCAGGGTC
F3H-Q-F	GGCACCATCACCCTCCTT
F3H-Q-R	CGATAGACGGCTGCTGTTC
F3′H-Q-F	CCTTCCTCCAAGCCATCA
F3′H-Q-R	GTCGCTACCTTTCACATCCA
F3′5′H-Q-F	GGTGGGAGTAGGAGTAGGGATTTT
F3′5′H-Q-R	AGTTGTGTCGGTTCCACCAATT
FLS-F	AGGAGGAGATTGCGGCTGTG
FLS-R	TAGTCGGTAGGGTTCTTGGG
DFR-Q-F	AACCTTTGTTGGATATTCGCG
DFR-Q-R	TCTTCTTGAACGTTCACCGTCC

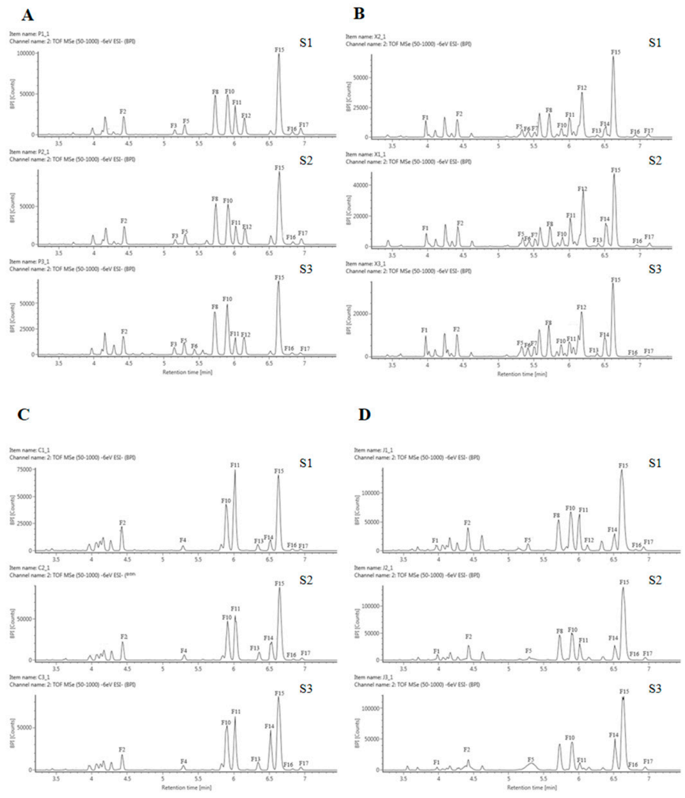

Figure S1. HPLC chromatogram of anthoxanthins in tepals at 3 flower development stages. (A)PM, (B) SL, (C)OE, (D)PD.

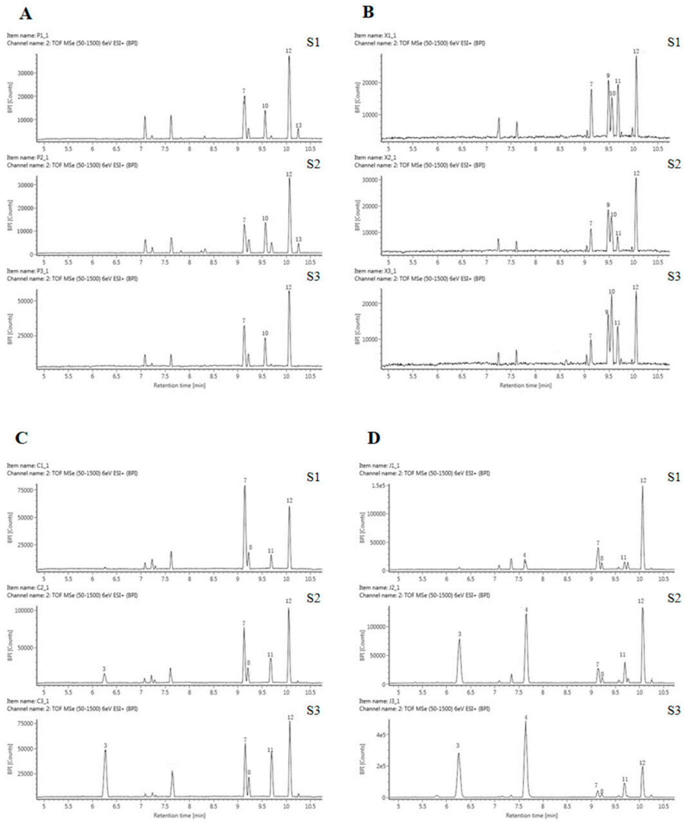

Figure S2. HPLC chromatogram of anthocyanins in tepals at 3 flower development stages. (A)PM, (B) SL, (C)OE, (D)PD.
